# Effect of Treadmill Exercise Timing on Repair of Full-Thickness Defects of Articular Cartilage by Bone-Derived Mesenchymal Stem Cells: An Experimental Investigation in Rats

**DOI:** 10.1371/journal.pone.0090858

**Published:** 2014-03-03

**Authors:** Jin-qi Song, Fu Dong, Xue Li, Chang-peng Xu, Zhuang Cui, Nan Jiang, Jun-jie Jia, Bin Yu

**Affiliations:** 1 Department of Orthopaedics and Traumatology, Nanfang Hospital, Southern Medical University, Guangzhou, Guangdong Province, China; 2 Department of Orthopaedics, Beihai People's Hospital of Guangxi Province, Bei Hai, Guangxi Province, China; 3 Key Laboratory of Bone and Cartilage Regenerative Medicine of Guangdong Province, Nanfang Hospital, Southern Medical University, Guangzhou, Guangdong Province, China; University of Pittsburgh, United States of America

## Abstract

**Objective:**

Current medical practice for the treatment of articular cartilage lesions remains a clinical challenge due to the limited self-repair ability of articular cartilage. Both experimental and clinical researches show that moderate exercise can improve articular cartilage repair process. However, optimal timing of moderate exercise is unclear. We aimed to evaluate the effect of timing of moderate treadmill exercise on repair of full-thickness defects of articular cartilage.

**Design:**

Full-thickness cartilage defects were drilled in the patellar groove of bilateral femoral condyles in a total of 40 male SD rats before they were randomly assigned into four even groups. In sedentary control (SED) group, no exercise was given; in 2-week (2W), 4-week (4W) and 8-week groups, moderate treadmill exercise was initiated respectively two, four and eight weeks after operation. Half of the animals were sacrificed at week 10 after operation and half at week 14 after operation. Femoral condyles were harvested for gross observation and histochemical measurement by O'Driscoll scoring system. Collagen type II was detected by immunohistochemistry and mRNA expressions of aggrecan and collagen type II cartilage by RT-PCR.

**Results:**

Both 10 and 14 weeks post-operation, the best results were observed in 4W group and the worst results appeared in 2W group. The histochemistry scores and the expressions of collagen type II and aggrecan were significantly higher in 4W group than that in other three groups (P<0.05).

**Conclusions:**

Moderate exercise at a selected timing (approximately 4 weeks) after injury can significantly promote the healing of cartilage defects but may hamper the repair process if performed too early while delayed intervention by moderate exercise may reduce its benefits in repair of the defects.

## Introduction

It is a great challenge for both orthopaedic surgeons and rehabilitation specialists to repair articular cartilage injuries or defects. Articular cartilage is a type of highly organized tissue with complex biomechanical properties and substantial durability,[1] but since it has a poor intrinsic capacity of self healing,[2] cartilage injuries usually lead to osteoarthritis, often ending in disabling symptoms.

Microenvironment plays an important role in maintenance and repair of articular cartilage.[3], [4] Creating an environment which can facilitate the healing process and simultaneously avoid potentially deleterious forces acting to the repair site is advantageous for cartilage repair process. It is known that degenerative changes of articular cartilage probably result from multifactorial reasons including nutritional deficiency.[5] Nutrition of chondrocytes occurs by diffusion, which depends on the viscoelastic properties of articular cartilage and movement of the joint. Compression and decompression forces during weight-bearing may nourish the articular cartilage.[6] It has been demonstrated that immobilization, even applied for a very short duration, exerts a harmful effect on healing articular cartilage [7], [8], [9]. Continuous passive motion has been reported to have beneficial effects due to the joint movement.[10], [11] Palmoski et al., however, found that joint motion in the absence of normal loading did not maintain normal articular cartilage and joint loading was more important to the biological and functional properties of normal articular cartilage.[12]

Exercise intensity may be an important influencing factor in cartilage repair. Studies have found that high-intensity exercise may lead to cartilage degeneration while moderate exercise can improve articular cartilage. [13], [14] Numerous studies have demonstrated that moderate running exercise improves the biological and biomechanical properties of articular cartilage.[14], [15], [16], [17] Even articular cartilage with degenerative changes may stand moderate exercise without showing further lesions[18], [19]. In vitro studies have also showed that higher levels of intermittent pressure may result in greater stimulation to collagen production [20], [21]. Some researchers found that moderate exercise can improve or at least not inhibit the cartilage repair.[22], [23], [24], [25]

However, some studies found that postoperative running did not enhance the healing of cartilage defects.[26], [27], [28] We hypothesized that the effect of exercise training on repair of cartilage defects might be also associated with different timing of exercise training intervention in addition to an association with exercise intensity. Few researchers have addressed the effect of timing of exercise training intervention on repair of cartilage defects. It is not clear when is the optimal time to start exercise training to facilitate spontaneous repair of cartilage repair, and there is great controversy about the timing of weight-bearing after articular cartilage injury in clinic.[29]

Therefore, we also hypothesized that exercise training of moderate intensity performed at a selected time may have a beneficial biological effect on the spontaneous healing of small full-thickness defects in articular cartilage. The purpose of our experimental investigation was to test the validity of this hypothesis, and to determine the optimal timing to initiate moderate exercise (treadmill training) after injury to promote the repair of small full-thickness defects.

## Results

### Macroscopic observation of cartilage defects

**Figure 1:**
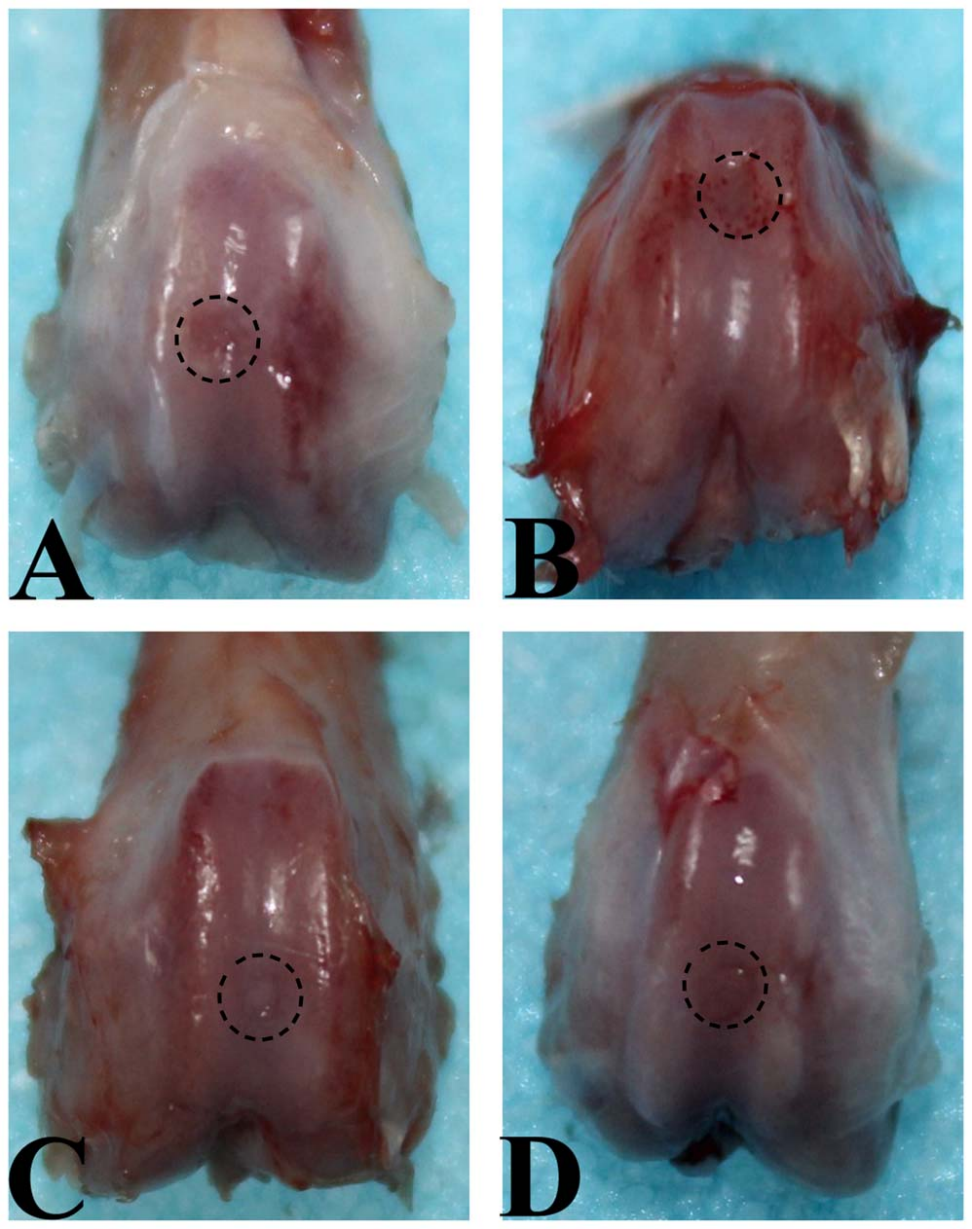
Macroscopic appearance of an operated knee at explantation after 10 weeks of follow-up. A. SED group. Defect is partially filled with repair tissue. B. 2W group. The margin of the normal cartilage around defect was eroded. C. 4W group. Defect is completely filled with white cartilage-like repair tissue to the level of surrounding uninjured cartilage, and the junction area is obvious. D. 8W group. Defect is filled with a white cartilage-like tissue with an irregular surface, and there is transparent line in the boundary area between the repair tissue and the residual cartilage.

**Figure 2:**
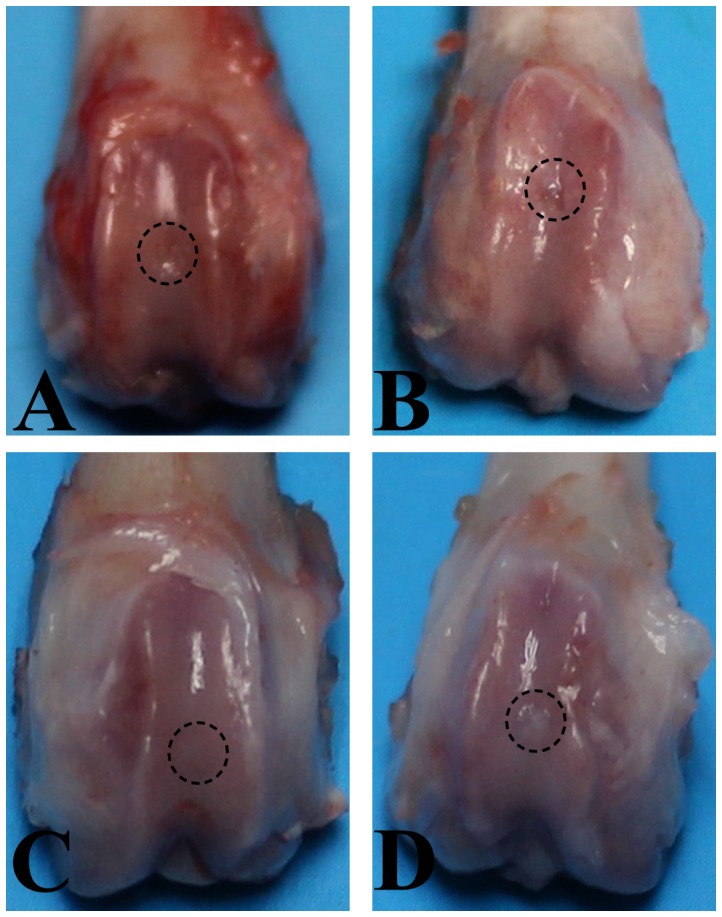
Macroscopic appearance of an operated knee at explantation after 14 weeks of follow-up. A. SED group. Defect is partially filled with repair tissue. B. 2W group. The margin of the normal cartilage around defect was eroded. C. 4W group. Defect is covered by smooth, glistening white tissue that was almost indistinguishable from the surrounding normal cartilage. D. 8W group. Defect is filled with a white cartilage-like tissue with an irregular surface, and the boundary of the defect was obvious.

Ten weeks after operation, none of the defects in the SED and 2W groups was completely filled with repair tissue. Instead they were partly filled with fibrous tissue (Fig. 1A–B). In 2W group, the margin of normal cartilage around the defect was eroded (Fig. 1B). Defects in 8W group were filled with a white cartilage-like tissue with an irregular surface, and the boundary area between the repair tissue and the surrounding original cartilage was obvious (Fig. 1D). In 4W group, better results were observed. The defects were completely filled with white cartilage-like repair tissue to the level of surrounding intact cartilage, and there was no cleft in the boundary though the repair tissue at the boundary was different from that at the center and the adjoining normal cartilage (Fig. 1C). After fourteen weeks, clear progress in repair was observed in all groups, but the repair process appeared to evolve a little faster in 4W group. The defects were covered by smooth, glistening, white tissue that was almost indistinguishable from the surrounding normal cartilage (Fig. 2C). There was no macroscopic manifestation of cartilage degeneration. However, the defects in SED and 2W groups were still partly filled and the surface of repair tissue was irregular (Fig. 2A–B). In 8W group, the defects were fully filled, the surface was almost flat, the repair tissue appeared like articular cartilage but different from the normal cartilage, and the boundary of defect was obvious (Fig. 2D). There were small clefts in the boundary area in some specimens in this group.

### Histological staining and semiquantitative histological findings

**Figure 3:**
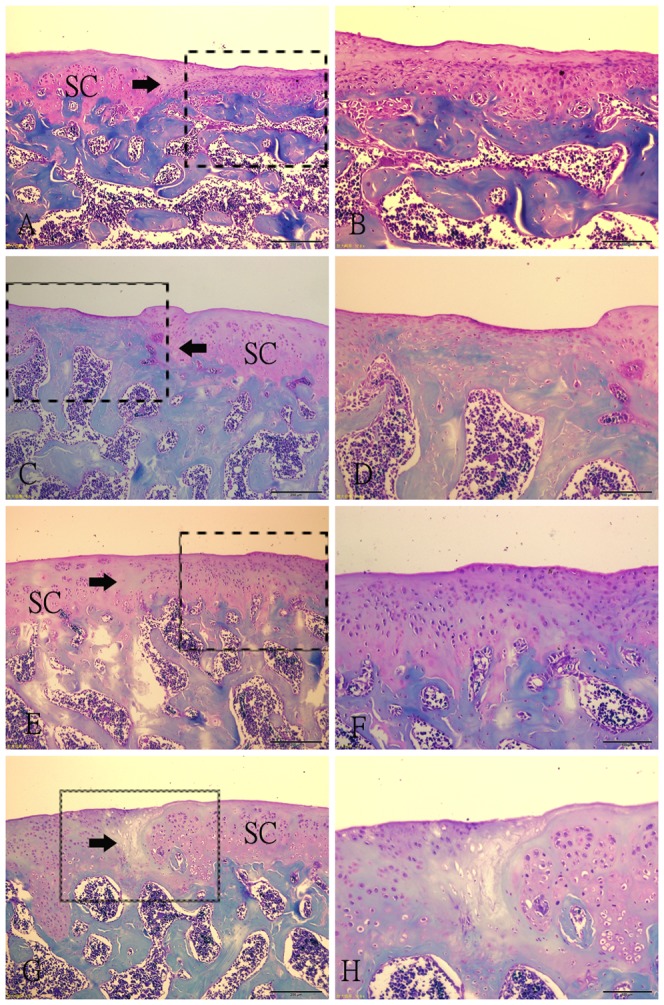
Histological photographs of the representative repair tissues of defects in four groups at 10 weeks after operation with safranin-O staining. (A–B) Fibrous tissue in SED group. The spindle-shaped fibroblasts embeded in thin fibrin-like tissue are present in the tissue. (C–D) Fibrous tissue in 2W group. The regenerative fibrous tissue is much thinner than that in SED group, and there are a little fibroblasts. The margin of the normal cartilage adjacent the defect is destroyed. (E–F) Repair tissue in 4W group. A large number of rounded cells embedded in the intensely Safranin-O stained extracellular matrix. The integration of the lesion to adjacent articular cartilage is good. (G–H) Regenerative tissue in 8W group. Some clusters of rounded cells resembling chondrocytes were recognized embedded in a fibrous and extracellular matrix. The bonding area is acellular. Fig. B, D, F, H (scale bar  =  200 µm) are higher magnifications of the junction of the repair tissue from Fig. A, C, E, G (scale bar  =  500 µm) respectively. SC: surrounding cartilage. →: defect margin.

**Figure 4:**
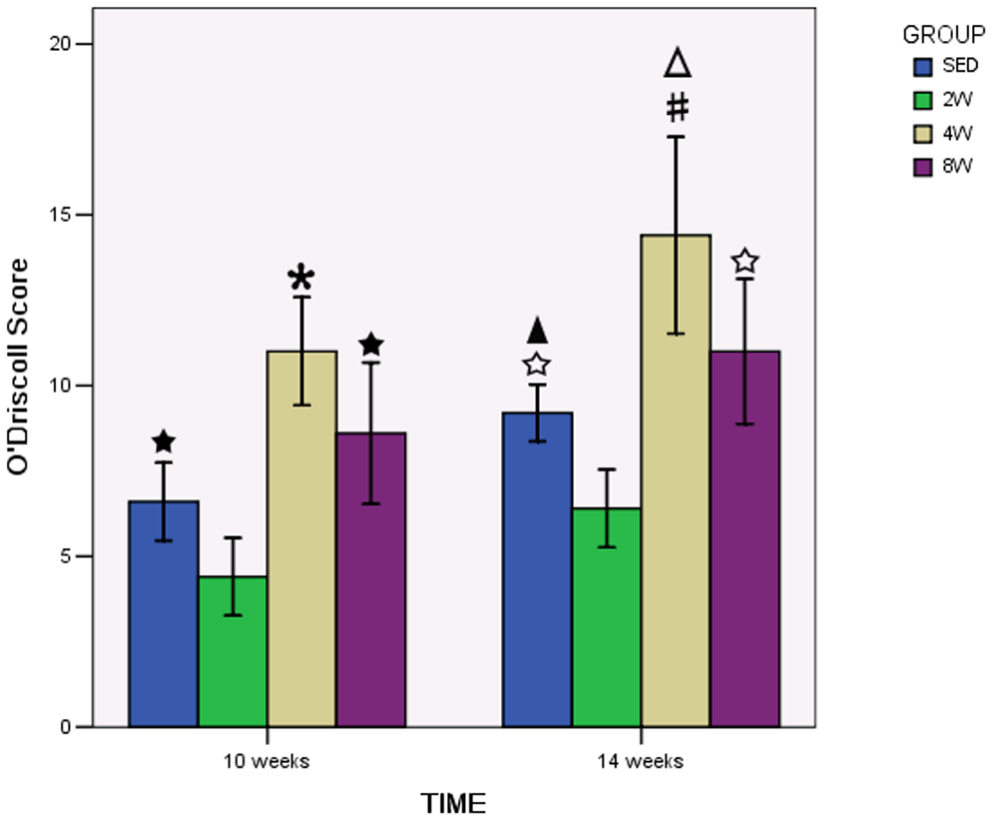
O'Driscoll score in each group. There was a significant increase in the 4W group compared to the to SED, 2W and 8W groups both in 10 weeks and 14 weeks after operation. * P<0.05 compared to SED, 2W and 8W group in 10 weeks after operation. #P<0.05 compared to SED, 2W and 8W group in 14 weeks after operation. ★ P<0.05 compared to 2W group in 10 weeks after operation. ☆ P<0.05 compared to 2W group in 14 weeks after operation. △P<0.05 compared to 4W group in 10 weeks after operation.▴P<0.05 compared to SED group in 10 weeks after operation.

Ten weeks after operation, sections demonstrated that repair tissue at the defect was more fibrous than fibrocartilaginous in SED and 2W groups (Fig. 3A–D). In these two groups, the thickness of the repair tissue was less than 50% of the normal cartilage, and the surfaces were irregular and covered with thin fibrin-like tissue, which contained spindle-shaped fibroblasts embedded in a poor matrix. The margin of normal cartilage adjacent to the defect was destroyed in 2W group (Fig. 3C–D). Sections from 8W group showed irregular surfaces and a thickness of regenerated tissue over 50% of the normal cartilage. Some clusters of rounded cells resembling chondrocytes were recognized embedded in a fibrous and extracellular matrix (Fig. 3G–H). Surface congruity and integration of the lesion to adjacent articular cartilage were better in 4W group. The regenerated tissue contained a large number of rounded cells, with a thickness similar to that of healthy surrounding cartilage. The extracellular matrix was intensely Safranin-O stained (Fig. 3E–F). The total score of semiquantitative histological analysis was significantly higher in 4W group (11.00±1.581) than in SED group (6.60±1.140), 2W group (4.40±1.140) and 8W group (8.60±2.074) respectively (Fig. 4). The average score in 2W group was significantly lower than in SED group, and the score in 8W group was similar to that in SED group.

**Figure 5:**
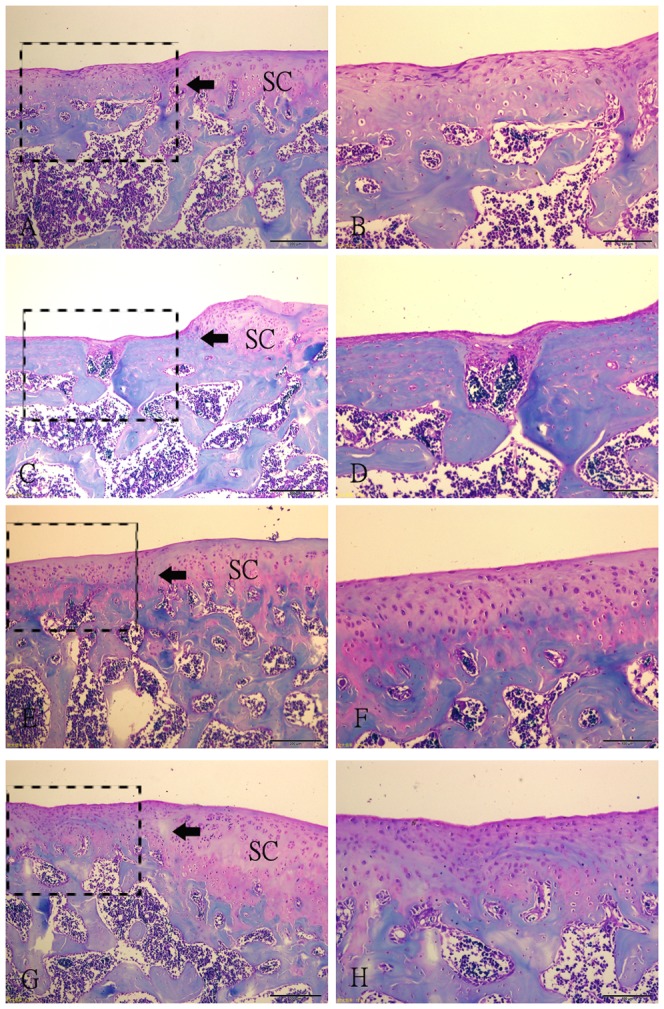
Histological photographs of the representative repair tissues of defects in four groups at 14 weeks after operation with safranin-O staining. (A–B) Fibrocartilaginous tissue in SED group. The extracellular matrix was poorly organized. (C–D) Fibrocartilaginous tissue in 2W group. The partly filled defect. The margin of the normal cartilage adjacent the defect is destroyed. (E–F) Repair tissue in 4W group. A large number of rounded cells embedded in the intensely Safranin-O stained extracellular matrix. The integration of the lesion to adjacent articular cartilage is good. (G–H) Regenerative tissue in 8W group. The surface of the repair tissue is irregular. The bonding area is acellular. Fig. B, D, F, H (scale bar  =  200 µm) are higher magnifications of the junction of the repair tissue and the residual cartilage from Fig. A, C, E, G (scale bar  =  500 µm) respectively. SC: surrounding cartilage. →: defect margin.

Fourteen weeks after operation, the fibrous tissue at the defect changed into fibrocartilaginous one in SED and 2W groups, but the defects were still partly filled and the extracellular matrix was poorly organized (Fig. 5A–D). In 4W and 8W groups, surface congruity was better compared with samples taken ten weeks after operation (Fig. 5E–H). However, bonding of the repair tissue to adjacent cartilage was still poor in 8W group. And we can see the tidemark of the repair tissue in some sections in 4W group (Fig. 5E–F). But the two groups had the same histologic appearance with regard to numerous densely distributed mature chondrocytes in the lacunae. No cartilage degeneration such as fibrillation, hypocellularity, and decreased proteoglycan was seen. The highest total score of semiquantitative histological analysis was obtained in 4W (14.40±2.881), and it was statistically significant difference in comparison to other groups (Fig. 4). The lowest score was found in 2W group, while score in 8W group was similar to score in SED group.

### Analysis of gene expression

**Figure 6:**
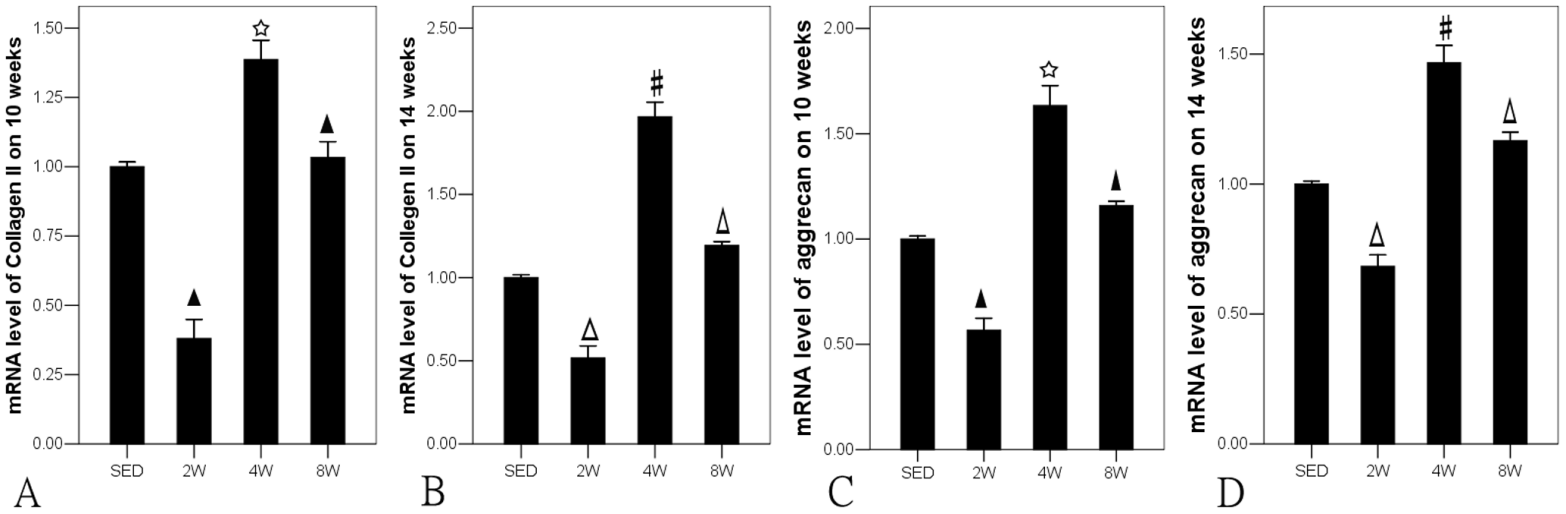
* P<0.05 compared to SED, 2W and 8W group in 10 weeks after operation. #P<0.05 compared to SED, 2W and 8W group in 14 weeks after operation. ★ P<0.05 compared to SED group in 10 weeks after operation. Δ P<0.05 compared to SED group in 14 weeks after operation.

Ten weeks after operation, the levels of collagen type II and aggrecan genes in 8W group were 3.09±0.65 and 1.24±0.46, respectively, significantly higher than those in SED (2.44±0.88 and 1.13±0.36, respectively) and 2W (0.94±0.62 and 0.51±0.36, respectively) groups (P<0.05), while the levels of collagen type II and aggrecan genes in 4W group (4.71±0.58 and 2.12±0.51, respectively) were significantly higher than those in the other three groups (P<0.05) (Fig. 6). Fourteen weeks after operation, the same situation was displayed among all groups, but the expression levels of aggrecan and collagen type II were elevated in all groups. In SED and 2W groups, the expression levels of collagen type II and aggrecan genes were still very low, though elevated compared with ten weeks after operation. The express levels in 8W group (5.60±0.57 and 1.73±0.79, respectively) were significantly higher than in SED (4.03±1.86 and 1.76±0.43, respectively) and 2W groups (2.07±1.07 and 0.69±0.43, respectively) (P<0.05), while lower than in 4W group (7.76±1.05 and 2.53±0.39, respectively) (P<0.05) (Fig. 6).

### Immunohistochemistry

**Figure 7:**
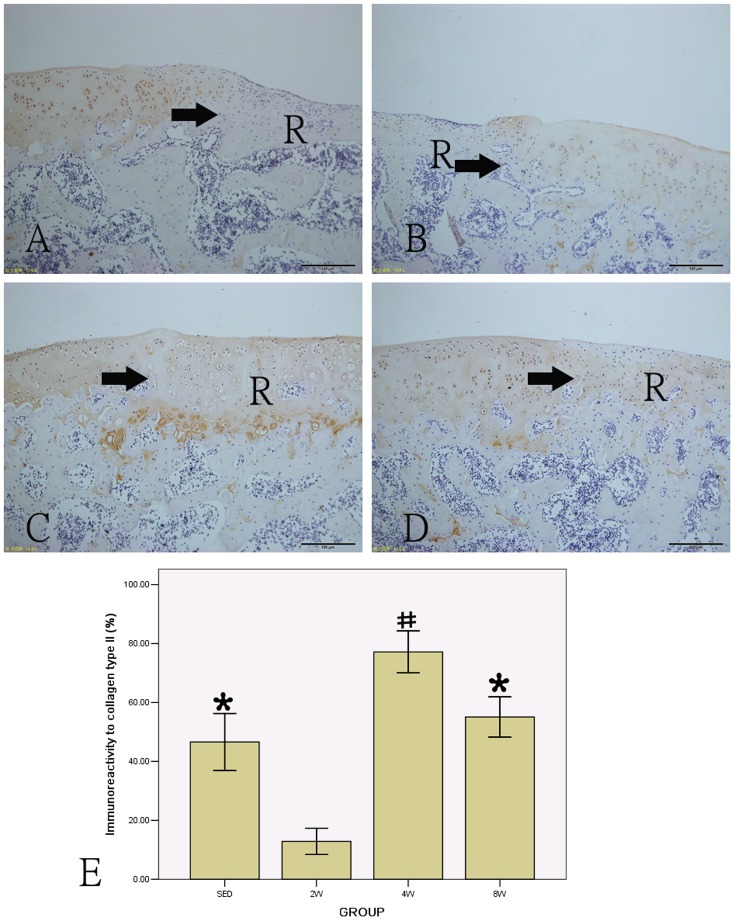
Immunohistochemical staining of collagen type II and the percentage of positive collagen type II in all groups (A: SED group; B: 2W group; C: 4W group; D: 8W group). # P<0.05 compared to SED, 2W and 8W group; * P<0.05 compared to 2W group. (scale bar  =  100 µm). R: repair tissue. →: defect margin.

Immunohistological analysis for collagen type II was performed in repair tissue sections in all groups at 14 weeks, and the content of collagen type II in each group is shown in Figure 7. There was a little collagen in 2W group (12.848±4.456). The collagen content in 8W group (55.088±6.813) was higher than in SED group (46.596±9.680), but there was no statistically significant (P>0.05). The collagen content in the 4W group (77.146±7.124) was significantly higher than in other groups (P<0.05), the difference was statistically significant.

## Discussion

In the present study, rats subjected to moderate exercise 4W following wounding had significantly better results in terms of morphology, histological scores, immunohistological values and gene expressions of collagen type II and aggrecan than those in other three groups (P<0.05). There was no significant difference between 8W and SED groups (P>0.05), and the results of 2W group were significantly inferior to those of SED group (P<0.05). The results of 8W group were significantly better than those of 2W group (P<0.05). In SED and 4W groups, the results of 14 weeks after operation were significantly better than those of 10 weeks (P<0.05). All these findings indicated that timing of exercise training remarkably affected the repair of cartilage defects. Exercise training initiated 4 weeks after operation remarkably promoted the spontaneous repair of cartilage defects, while training initiated 2 weeks postoperatively did more harm than good, and that initiated 8 weeks after surgery had insignificantly beneficial impact on normal cartilage repair.

At 10^th^ and 14^th^ weeks after operation, 2W group had the minimal spontaneous repair tissue at the defects with the surrounding cartilage damaged in most specimens. And all the results of this group were significantly poorer than those of SED group (P<0.05). This, we think, was attributed to the forces from the exercise training onto the cartilage defect which had been introduced too early in the healing process and interfered with the clot which was required for the modulation of mesenchymal cells from the subchondral bone to produce regenerative tissue [30]. This indicates that exercise training initiated at 2 weeks postoperatively in a rat model may be too early to be beneficial for the cartilage repair. This also indicates that a joint loading too early may have a deleterious impact on cartilage repair. This finding of ours is consistent with one well-known principle of articular cartilage rehabilitation which involves weight-bearing restrictions at the early process. [31]

The current study demonstrated that all the results of 4W group at 10 and 14 weeks were significantly better in terms of promoting cartilage repair than that in the other two exercise training groups and the non-training group as well (P<0.05). It is noticeably consistent with the previous reports [32], [33], [34] which demonstrated that exercise training enhanced the contents of collagen type II and aggrecan in repair tissue. However, timing of exercise training was not a concern in these studies. Our study was concerned with the time issue and found that exercise training initiated only at 4 weeks postoperation had a beneficial effect on repair of small cartilage full-thickness defect in a rat model. What is more, the positive effect could sustain as the exercise continued for the four week group.

Results of 8W group indicate that exercise training initiated too late may render no benefit to the spontaneous cartilage repair but may be better than an exercise training initiated too early which is harmful to cartilage repair. At 8 weeks in the cartilage repair process in a rat model, new cartilage-like tissue has been generated, and the benefits of exercise training initiated at this time point may be diminished.

With regard to the intensity of exercise, a number of previous studies have shown that, excessive exercise had detrimental effect on articular cartilage [14], [17], [35], [36]; low intensity exercise had little effect on articular cartilage[17], while moderate intensity exercise improved the biological and biomechanical properties of articular cartilage[15], [16], [17]. On the basis of the previous findings, we chose the same intensity and frequency of exercise during the entire intervention period in order to ensure the homogeneity of the intervention factors. However, from a clinical perspective, both the timing and intensity of the exercise intervention are expected to be adjusted in the treatment protocols. For example, exercise would be initiated at a low intensity level, and then gradually progressed to more intense levels over time. Moreover, even different modes of exercise might also have a potential effect on the cartilage repair. The effects of different compositions of different timings intensities and modes on the outcomes of cartilage repair are too complicated to be addressed in the present comparatively simple experiment. Further research should be performed to investigate the composite effects of likely influencing factors of cartilage repair, particularly time versus different intensity interaction.

In literature regarding exercise training, exercise intensity was usually evaluated by maximum oxygen consumption (VO_2 max_). In Laforgia's [37] report, low intensity was defined as less than 50% VO_2max_, moderate intensity as 50–75% VO_2max_ and high intensity as more than 75% VO_2max_. The standard for running of moderate intensity we used was recommended by Bedford et al [38]. They developed a running test for rats in which they standardized maximum oxygen consumptions of five modes of running. Running for 40 min on a 10° grade treadmill at a speed of 19.3 m/min was equated the maximum oxygen consumption of 75% and named as physiological level exercise which means exercise of moderate intensity in terms of Vo_2 max_. In the present experiment, the rats were subject to exercise of moderate intensity, namely, running for 40 min on a 10° grade treadmill at a speed of 19.3 m/min.

One limitation of the present investigation is that it offers no insight as to potential mechanisms by which the initiation of exercise at different time points after injury may have differing effects on cartilage healing. In fact investigation into the potential mechanisms is a challenge that needs intensive efforts. However, the following may explain the possible mechanisms according to the previous relative findings.

In vitro studies demonstrated that mechanical stimulation to MSCs for a period of pre-differentiation enhanced chondrogenic differentiation of the MSCs. For example, Huang AH et al. found that loading initiated immediately following construct creation elicited negative effects on DNA, sGAG and collagen content, while loading initiated after 3 weeks of chondrogenic preculture not only abrogated these negative effects but also improved the functional properties and extracellular matrix distribution.[39] They attributed these different responses to loading to changes in MSC phenotype. Similarly, another study concluded that the differentiation status of MSCs might play an important role in response to mechanical stimulation as compared with differentiated cells because the nuclei of undifferentiated stem cells might deform more readily to mechanical stimulation. [40] In other words, different differentiation status of MSCs means different durations of differentiation of MSCs after culture. In our experiment, moderate exercise initiated at 4 weeks after injury resulted in the best effect on repair of defected cartilage. A possible explanation for this might be that the neoformative tissue was at a suitable time point to perceive mechanical stimulation.

We found that exercise initiated 8 weeks after operation had insignificantly beneficial impact on normal cartilage repair. This may be contributed to cell-matrix interactions. A recent study demonstrated that cell-matrix interactions might also affect load-induced response but only after MSCs differentiate and generate local extracellular matrix (ECM). In pace with matrix elaboration, the physical environment of the cells under mechanical stimulation might be also altered [39]. At 8 weeks after injury, the MSCs of the neoformative tissue might have differentiated and generated local ECM so that the cell-matrix interaction might have changed the appropriate physical environment of the cells for mechanical stimulation.

Another potential mechanism might be explained by facilitated nutrient transport with dynamic deformation. Nutrition of the chondrocytes occurs by diffusion, which depends on the viscoelastic properties of articular cartilage and the movement of the joint. Compression and decompression forces during weight-bearing nourished the articular cartilage[6]. Recent experimental findings showed that the transport of large solutes was facilitated by dynamic compression [41], [42]. Exercise might provide dynamic stimulation to facilitate the nutrient transport necessary for cartilage growth. Regretfully, no research has been conducted on the timing of dynamic stimulation on the nutrient transport or on the nutrient transport of the growing cartilage at different periods after injury.

The rats we used were 8 weeks old. Rats reach sexually mature at 6 weeks of age, but become social maturity at about age 5 to 6 months.[43] It is generally recognized that articular cartilage chondrocytes from social immature animals have a greater capacity to proliferate and synthesize larger proteoglycan molecules.[44] The different effects of mechanical to cartilage repair of mature and immature animals is still unknown, and further research should be performed to ascertain the differences.

Our experiment may provide some evidence or insight for practical clinical problems. For instance, timing of weight-bearing after articular cartilage injury in clinic is controversial. Our study demonstrated that proper exercise training performed at a selected time is beneficial to cartilage repair, but may be harmful if initiated too early and almost useless if too late. Likewise, weight-bearing should be conducted in a similar manner. It deserves intensive research whether proper weight-bearing performed at a selected time is beneficial to cartilage repair.

A well accepted mechanism of cartilage repair is that mesenchymal cells from the subchondral bone can produce regenerative tissue after injury. Bone marrow stimulation techniques such as microfracture are commonly used as the first line treatment for cartilage lesion. Mechanical stimulation also has an effect on cartilage repair. Pain relief and functional improvement are observed after moderate exercise has been encouraged as an adjunct rehabilitation of cartilage lesion in clinic.[45] Although mechanical stimulation has a potential to induce mesenchymal stem cell chondrogenesis, [21] there is no evidence that it can increase the concentration of MSCs from subchondral bone. Therefore, exercise alone cannot be used as a major therapy for cartilage defects in clinic. It can be applied as an adjunct to microfracture, or other techniques that involve breaching the subchondral bone plate to initiate repair in clinical rehabilitation. Further studies are necessary on the effects of exercise combined with other techniques on cartilage repair.

In our study, the histological staining images showed that the cartilage surrounding the drill site displayed chondrocyte cloning and had areas of accelularity, indicating that, to some extent, the cartilage was osteoarthritic.[46] This indicates that cartilage defects can make the whole articular facet vulnerable. This may be a reason why a serious cartilage defect cannot be repaired simply by mechanical stimulation or exercise of great intensity. So further research should be performed on the relationship between exercise intensity and the final outcomes of joint with cartilage defect before we can have a better understanding of the effects of exercise on articular cartilage repair.

Our study explored the selected timing for exercise training, but the results should be interpreted with caution. Firstly, 4 weeks after operation is an approximately selected time point that led to the best result but not an exactly optimal time to initiate exercise training. Next, our results were suitable only to our present model of cartilage defect. Different kinds of cartilage defect, different animal models and different intensities of exercise training will produce different results. Cartilage lesions in clinic are various and consequently repair strategies should also vary, including the role of exercise training. The effects of exercise training should also depend on the variety of cartilage defects. Similarly, the size and severity of a cartilage defect will greatly affect the role of exercise training in the repair process. Thirdly, although the results showed that exercise initiated four weeks after operation had a better impact than exercise initiated two or eight weeks after operation, and better than absence of exercise, the disparity between the repair cartilage and normal articular cartilage was unclear. Further research should be performed to investigate the difference in order to have a comprehensive understanding of the effects of exercise on articular cartilage repair. Besides, though the PCR technique has the characteristics of high specificity and high sensitivity, the addition of more quantitative measures of cartilage healing will corroborate the results.

Of course, besides all these important variable in the research of cartilage repair problems, timing of intervention is always a problem we cannot ignore. The most noteworthy finding of the present study is that timing plays an important role in moderate exercise applied as an adjunct to other treatments in clinical rehabilitation for cartilage lesions.

## Materials and Methods

### Experimental animals and study protocol

A total of 40 mature male Sprague Dawley rats (8 weeks old, weighing 200 to 250 g) were housed in cages under controlled light/dark (12/12 h) and temperature (22 ± 1°C) conditions and provided with food and water ad libitum. The animals were allowed to get adapted to running for one week before they received operation.

**Figure 8:**
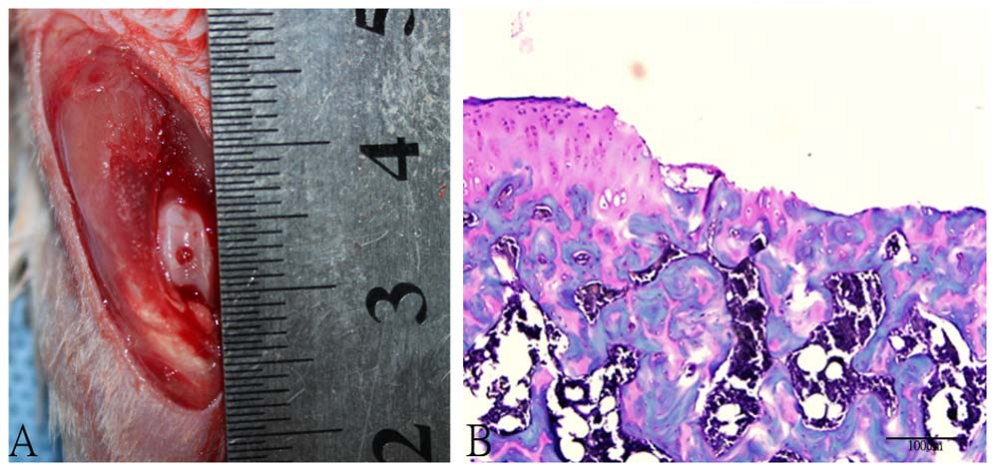
The full-thickness defects of patellofemoral articular surface. A. Gross appearance of full-thickness articular surface defects. B. Safranin O-Fast Green staining of articular surface defects. (Original magnification × 200).

Small full-thickness cartelage defects were created at the bilateral knee joints in each animal using a standard operative procedure previouly described [47] under general anaesthesia (sodium pentobarbital 0.2 m1/100 g body weight, i.p.). The level of anaesthesia was controlled through the plantar and pupillary reflexes. After skin shaving and disinfection, a medial parapatellar incision was made to dislocate the patella laterally. The joint was temporarily flexed to expose the femora1 trochlea. Perforation of the articular cartilage at the middle of the femoral trochlea, 2 mm from the intercondylar fossa, was made with the same hand drill (diameter 1.0 mm) until bleeding (Fig. 8) when the drill reached the subchondral bone and the resident tissue of the subchondral bone was preserved. The joint was washed with a sterile saline solution (0.9% NaCl) to remove the cartilaginous and osseous debris. The medial capsular incision (nylon 6/0) and the skin (silk thread 4/0) were sutured before the skin was disinfected with polyvidone-iodine. Antibiotics (penicillin 0.04 m1/100 g body weight, i.m.) was given to the rats after operation. All of the surgeries were performed by the same investigator (JQ.S.).

Next the rats were randomly and evenly assigned to one of the four groups as follows: 1) sedentary control group (SED) in which the rats were kept in their cages where they could move freely at will until sacrifice. 2) 2W group in which the rats got running training 2 weeks after operation, 3) 4W group in which the rats started running training 4 weeks after operation and 4) 8W group in which the rats got running training 8 weeks after operation. The running intensity was moderate, determined according to the maximum of oxygen consumption, as recommended by Bedford.[38] The animals were made running for 40 min on a 10° grade treadmill at a speed of 19.3 m/min, once per day and *5* days a week.

The rats were euthanized for sampling with an overdose of sodium pentobarbital. Samples of the knee joint were harvested ten (n = 5, respectively) and fourteen weeks (n = 5, respectively) after creation of the full-depth articular cartilage defects. The hindlimbs were amputated and the knee joints were immediately opened and disarticulated. At each time point, in evry group, 5 knees were used for histomorphological and immunohistochemistry evaluations while the other 5 knees for PCR evaluation.

The experiment was approved by the animal ethics committee of the Nanfang Hospital.

### Sample preparation

Photographs of the samples were taken with a single-lens reflex camera for morphologic obsevation while keeping the articular surfaces moistured with sterile physiological saline solution.

For immunohistochemistry and histological examinations, femoral condyles on the right sides in each group were dissected and fixed immediately in fixative solution (4% paraformaldehyde in 0.1 M phosphate buffer, pH 7.4) for 24 hours. Decalcification was completed in 10% EDTA solution before the samples were embedded in paraffin wax. Thereafter, they were cut into 1-mm sagittal sections in the medial region. From each block histological sections were cut perpendicularly to the repair tissue and prepared systematically: the first four sections (5-pm-thick) were stained with Safranin O, and the next two sections were used for immunohistochemistry.

For RT-PCR analysis, samples of repair articular cartilage from femoral condyles on the left sides were obtained with a drill. In order to ensure that the samples were harvested exactly from the defect zone, the harvest area was determined using the same method as used in creating the defect models. It was at the middle of the femoral trochlea, 2 mm from the intercondylar fossa. The harvested samples were cylindrical tissues from the defect area, with 1 mm in diameter and about 0.2 mm in height. The samples were flash-frozen in liquid nitrogen at −80°C.

### Histomorphological evaluation

The samples were stained with Safranin-O and histomorphologically evaluated with O'Driscoll scoring system (Table.1) [10]. Parameters used were the following.

1. Nature of the predominant tissue: (1) cellular morphology (maximum, 4.0 points).
2. Structural characteristics: (1) surface regularity (maximum, 3.0 points), (2) structural integrity of the repair tissue (maximum, 2.0 points), (3) thickness of the repair tissue (maximum, 2.0 points), and (4) bonding of the repair tissue with the host cartilage (maximum, 2.0 points).
3. Freedom from cellular changes of degeneration: (1) degree of cellularity of the repair tissue (maximum, 3.0 points) and (2) chondrocyte clustering of the repair tissue (maximum, 2.0 points).
4. Freedom from degenerative changes in adjacent cartilage: (1) cellular characteristics in adjacent cartilage (maximum, 3.0 points) and (2) the presence of structural degenerative changes (modified), that is, fibrillation (maximum, 3.0 points).

**Table 1:** O'Driscoll Score system [10].

Criterion	Score
**Nature of predominant tissue**	
Cellular morphology	
Hyaline articular cartilage	4
Incompletely differentiated mesenchyme	2
Fibrous tissue or bone	0
Safranin O staining of matrix	
Normal or nearly normal	3
Moderate	2
Slight	1
None	0
**Structural characteristics**	
Surface regularity	
Smooth and intact	3
Superficial horizontal lamination	2
Fissure—25 to 100→ of the thickness	1
Severe disruption, including fibrillation	0
Structural integrity	
Normal	2
Slight disruption, including cysts	1
Severe disintegration	0
Thickness	
100→ of normal adjacent cartilage	2
50 to 100→ of normal cartilage	1
0 to 50→ of normal cartilage	0
Bonding to the adjacent cartilage	
Bonded at both ends of graft	2
Bonded at one end, or partially at both ends	1
Not bonded	0
**Freedom from cellular changes of degeneration**	
Hypocellularity	
Normal	3
Slight	2
Moderate	1
Severe	0
Chondrocyte clustering	
No clusters	2
<25→ of the cells	1
25 to 100→ of the cells	0
**Freedom from degenerative changes in adjacent cartilage**	
Normal cellularity, no clusters, normal staining	3
Normal cellularity, mild clusters, moderate staining	2
Mild or moderate hypocellularity, slight staining	1
Severe hypocellularity, poor or no staining	0

A total indices of healing score were derived by the summation of categories 1 through 4, and the maximum value was 24.0 points. The sections were graded by two observers that were kept unaware of the groups.

### Immunohistochemistry for collagen type II

In addition to histomorphological evaluation, immunohistological analysis for collagen type II was performed in all sections. After deparaffinization and rehydration of the tissue sections, collagen type II was immunostained with the two-step immunohistochemistry method instructed by the manufacturer (Zhongshan Goldenbridge Biotechnology Co., Ltd, Beijing, China).

The sections were incubated with monoclonal mouse antirat collagen type II antibody (1:200 dilution, Fisher Scientific, Chicago, IL, USA) for 3.5 h at 29°C. The slides were washed in PBS three times, and followed by a 20-minute incubation at 37°C with anti-mouse IgG/HRP (Fisher Scientific) and visualized with DAB chromagen. The slides were stained for 40 s before the nuclei with hematoxylin were counterstained for 6 s. The collagen type II content was evaluated based on optical density measured using image analysis software (Nikon H600L Microscope and Image-Pro Plus 6.0 image analysis system).

### Gene expression analysis by quantitative reverse transcription-polymerase chain reaction (RT-PCR)

The total RNA from the regenerated tissue was isolated using Trizol (Life Technologies Co., Ltd. Carlsbad, California) according to the manufacturer's protocol. The primer sequences specific for the target gene and the internal control gene [glyceraldehyde-3-phosphate dehydrogenase (GAPDH)] used for qRT-PCR are listed in Table 2. Briefly, the mRNA was converted to cDNA, and the realtime PCR was performed in an ABI 7500 (Applied Biosystems) with One Step SYBR_ PrimeScript_ RT-PCR Kit (TaKaRa Biotechnology Co., Ltd. Dalian, China) under respective conditions, and the fluorescence intensity was recorded for 40 cycles. The expressions of collagen type II and aggrecan target gene were analyzed. All assays were run in triplicate, and the specificity of amplification was controlled by no template and melting curve analysis. The Quantitation-Comparative Ct method was used to calculate the relative expression of each target gene.[48], [49]

**Table 2:** Primer sequence and product length of Collagen type II and aggrecan genes.

Target gene	Sequence	Product length
Collagen	5′- GAAGGATGGCTGCACGAAAC -3′	89 bp
	5′- CAACAATGGGAAGGCGTGAG-3′	
Aggrecan	5′- ACATCCCAGAAAACTTCTTT -3′	86 bp
	5′- CGGCTTCGTCAGCAAAGCCA -3′	
GAPDH	5′- AGCCGCATCTTCTTGTGCAGTG -3′	96 bp
	5′- TGGTAACCAGGCGTCCGATACG -3′	

### Statistical analysis

Results were expressed as the mean ± standard deviation. Data from semiquantitative histological analysis were analyzed by Fisher's exact test. Statistical analyses were carried out using a 2-way ANOVA. P<0.05 denoted the presence of a significant difference between groups.

## Conclusion

The present study demonstrates that a moderate exercise protocol may be effective for small full-thickness cartilage repair, shortening recovery time and enhancing the healing process, only when it is started at a selected time. Exercise training performed too early may be destructive to the repair tissue while that performed too late do not significantly promote the healing. Further experiments are necessary to reveal the mechanisms involved and to address whether moderate postoperative exercise can result in long-term improvements in cartilage repair.
